# Saline Stress Impairs Lipid Storage Mobilization during Germination in *Eruca sativa*

**DOI:** 10.3390/plants12020366

**Published:** 2023-01-12

**Authors:** Emilio Corti, Sara Falsini, Silvia Schiff, Corrado Tani, Cristina Gonnelli, Alessio Papini

**Affiliations:** Department of Biology, University of Florence, Via Pier Antonio Micheli 1–3, 50121 Firenze, Italy

**Keywords:** salt stress, *Eruca sativa*, seedling development, lipid reservoirs, cell ultrastructure

## Abstract

Soil salinization become worse in the last decades, leading to reduced crop yields, especially in the Mediterranean basin. Eruca sativa is a common species cultivated in this area with remarkable economic importance. This study aimed at investigating the effect of salinity on this plant, focusing on (i) seedling development in terms of variations in germination and growth parameters and (ii) anatomical and ultra-structural changes in the morphology of cotyledons. For this reason, seeds were treated with different salinity levels ranging from 137 to 548 mM NaCl. Seed germination was delayed by all the concentrations tested, but only above 137 mM seedling growth was impaired. Results showed a high occurrence of lipid bodies within the mesophyll cells of cotyledons of seedlings exposed to salt concentrations above 137 mM, suggesting an impairment in lipid mobilization caused by salinity during plant development. The cotyledons of treated seedlings showed reduced intercellular spaces and ultrastructural changes in chloroplasts and peroxisomes. Moreover, salt-induced autophagic processes were present in samples grown at the highest NaCl levels. Interestingly, at 137 mM NaCl, seedlings showed the highest values of mesophyll thickness and fresh weight, implying a possible mechanism of salt adaptation during germination.

## 1. Introduction

Salinization is the increase in concentration of soluble salts of sodium, potassium, magnesium, and calcium in the soil, which eventually might cause the reduction of its fertility with severe consequences for the ecosystem [1]. A soil can be defined as “saline” when the salt amount leads to an electrical conductivity of 4 dS m^−1^ (equal to 40 mM NaCl) or more [2].

Soil salinization can be the result of natural conditions or human activities. Natural reasons comprise weathering of rocks or salt deposits due to precipitation [3], while anthropogenic causes of soil salinization involve irrigation with brackish or wastewater containing high amount of minerals, or perturbation of hydrogeological configurations [4]. 

The salinization of soil heavily affects agriculture due to the decline in crop yield [5]. Salinity has two main effects on plants: osmotic stress at early steps, and ionic stress after longer periods [6]. Salt stress limits plant growth [3], leaf expansion, and primary carbon metabolism due to nutritional imbalance, water deficit, and osmotic and oxidative stresses [7]. To cope with such a stress situation, plants can accumulate organic solutes as carbohydrates (sucrose, trehalose) and nitrogen compounds (proline, betaine, glycine) as well as inorganic ions such as Na^+^ and K^+^ to lower cellular osmotic potential [8]. Moreover, they can reduce the number of aquaporins (water channels) in the membranes and thus diminish the hydraulic conductivity [9]. Instead, to counteract oxidative stress, plants resort to antioxidant substances as for instance ascorbate and carotenoids, along with detoxifying enzymes such as superoxide dismutase and catalase [10]. Regarding the developmental stages, salt negatively impacts seed germination and seedling development, thus influencing the vitality of the adult plants [11,12,13]. In particular, salt stress negatively affects seed germination both osmotically, through water absorption reduction, and ionically, through Na^+^ and Cl^−^ accumulation, causing a disorder in nutrient uptake and toxicity effects [14,15,16]. In fact, Na^+^ and Cl^−^ ions can negatively interfere with many biochemical processes and can impair lipid reserve mobilization during germination that is a fundamental process for the subsequent seedling development [17,18,19]. Thus, ultrastructural changes occurring in lipid mobilization during seedling development in salt stress conditions were evidenced [17,20]. In both studies, salts impaired the normal lipid mobilization of the lipid reservoir, since in salt-affected seedlings, the authors noted a higher occurrence of lipid bodies within the cotyledons. Considering that lipid body reservoirs are dynamic structures that are present in the seedling cells for a finite period of time, they can be considered a suitable marker to demonstrate a regular development of the plant and can be a tool to understand the seedling response against adverse conditions [20]. 

In addition, Cui et al. (2016) [21] reported the crucial interaction between peroxisome and oil bodies for lipid reservoir mobilization in Arabidopsis that is regulated also by environmental signal. Given this, investigating the occurrence of a physical interaction between peroxisomes and lipid bodies under salinity could be important to understand the lipid bodies mobilization in stress conditions. 

Moreover, salt stress also affects the anatomy and the cell structure of plant at every developmental stage [10]. Regarding the anatomy of leaves, changes in mesophyll thickness, intercellular space, mesophyll cell dimension, and epidermal thickness was observed in plants species under salinity conditions, showing different responses depending on species and salt tolerance capability [22,23,24]. Regarding the ultrastructure, it is well known that salinity induces changes in several components of plant cells, especially in chloroplasts, mitochondria, and plasma membrane, and it can also induce autophagic activity [10,25].

Since salinization of soil is becoming worse in countries of the Mediterranean basin [26], studies on the effect of salt stress on plants cultivated in this area have become crucial. *Eruca sativa* Hill (or *E. sativa* Miller or *E. vesicaria* (L.) Cav. Ssp. *Sativa* (Miller) Hegi), also known as arugula or rocket, is an annual herbaceous species that grows in the Mediterranean region and is widely cultivated, especially in the vicinity of coastal areas. Rocket leaves are commonly eaten raw, accompanying a large number of local dishes, and for this reason, its cultivation has a remarkable economic importance in many coastal regions [27]. Regarding seed germination of *E. sativa* under salt stress conditions, Miceli et al. (2003) [28] recorded a germination percentage of 90% under salt stress up to 10 mS cm^−1^ with no remarkable morphological variation in seedling plants. Fallahi et al. [13], testing the germination of *E. sativa* under different salinity levels, observed a reduction of the germination rate by 16% and 29% under 100 and 150 mM of NaCl, respectively. In addition, the authors showed a germination percentage of 38% for *E. sativa* under salinity stress of 200 mM. Other plant species as *Brassica oleracea*, *Cynara scoolymus,* and *Echinacea purpurea*, subjected to the same salt stress level, showed germination percentages of 5%, 6%, and 2%, respectively [29,30], thus showing the appreciable salt tolerance of rocket. A similar trend of *E. sativa* germination was observed by mathematical models in which Bakhshandeh et al. [31] estimated for *E. sativa* a salinity threshold of 257 mM NaCl for seeds and 247 mM NaCl tolerance threshold for seedlings, on the basis of a 50% reduction of seed germination and normal growth seedling percentage.

Despite the wide literature about several aspects of *E. sativa* affected by salt excess at various development stages, studies on the anatomy and ultrastructure of *E. sativa* seedlings under salinity stress are still missing. Moreover, although lipids are the main energy source for seedling development after germination in oil seeds plants as rocket [16,32], no microscopy studies about lipid storage mobilization in salinity conditions on *E. sativa* have been performed. Obtaining this information could help to understand the response of *E. sativa* under salinity in order to set strategies aimed to improve the tolerance of the species against this abiotic stress.

In the light of above, the aims of this paper were (i) to investigate how salinity affected seedling development, considering the growth parameters, and (ii) to provide, for the first time, anatomical and ultrastructural observations of the changes of both tissues and cells cotyledons in the cultivated plant of *E. sativa*. In particular, the study focused on seedling lipid reservoirs, hypothesizing the detrimental effect of salt on the mobilization of this source of energy during the first stages of plant development. 

## 2. Results

### 2.1. Germination Analysis

**Figure 1:**
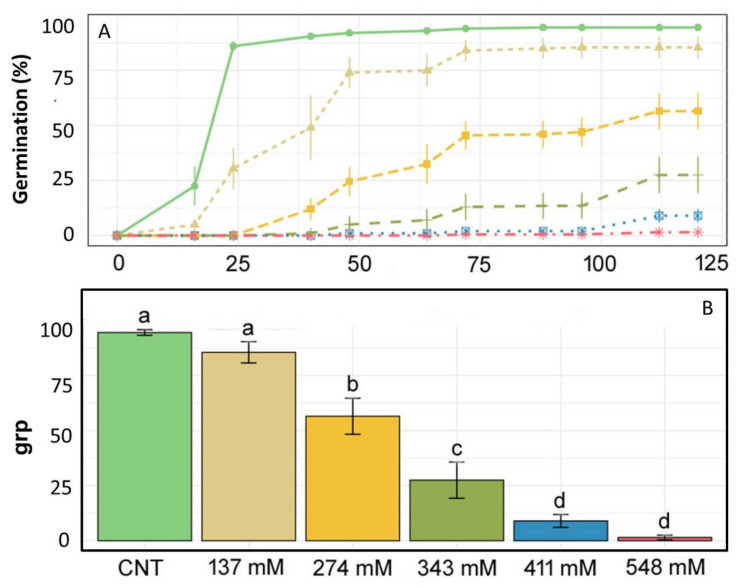

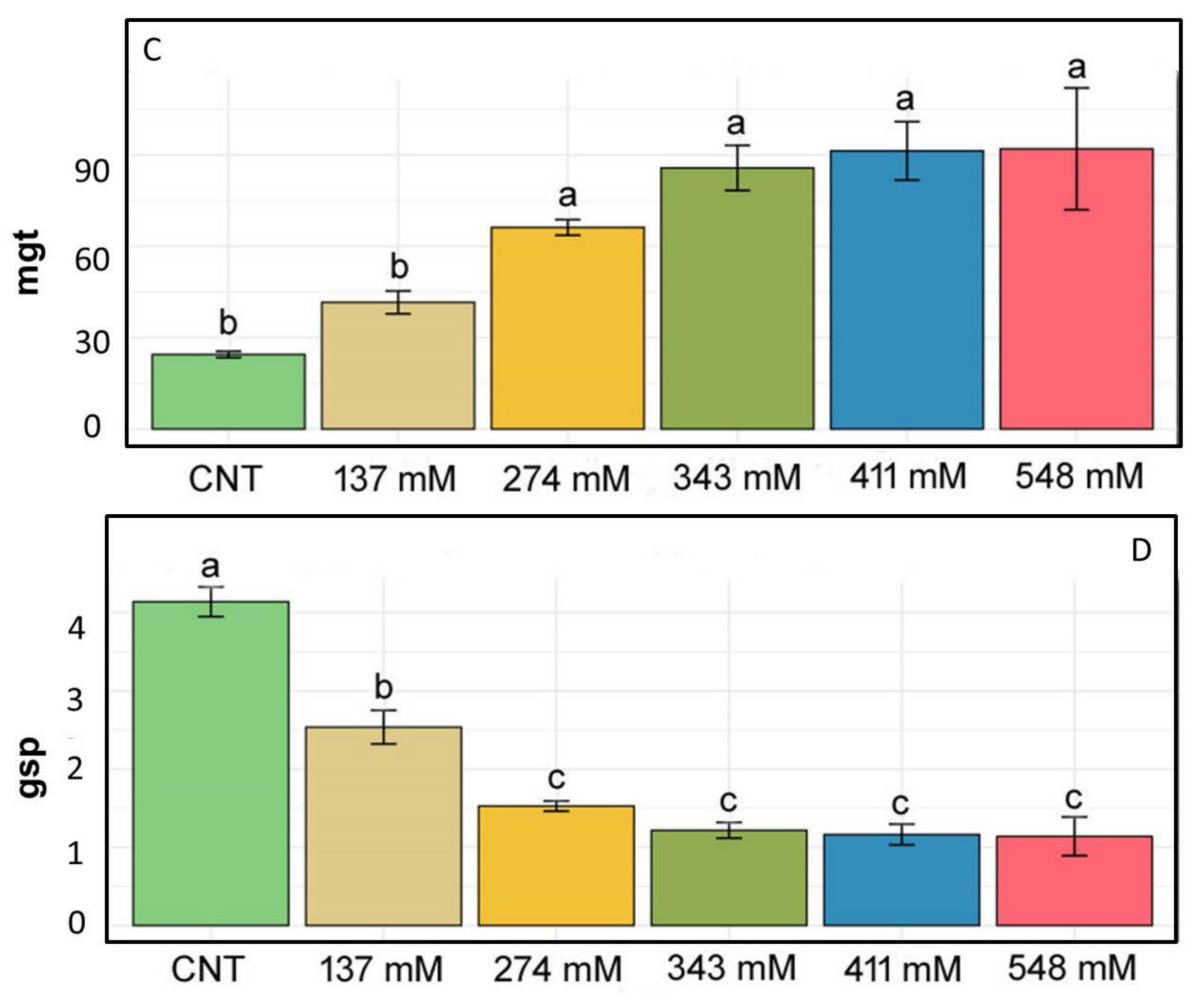
(**A**) Germination lines, (**B**) germination rate percentage (GRP), (**C**) mean germination time (MGT), and (**D**) germination speed percentage (GSP) for *E. sativa* given various treatments: only distilled water as a control (light green), 137 mM NaCl (light brown), 274 mM NaCl (yellow), 343 mM NaCl (dark green), 411 mM NaCl (blue), 548 mM NaCl (pink). Different letters above bars indicate significant differences (*p* < 0.05) among treatments.

The effect of different NaCl concentrations on the germination of *E. sativa* is shown in Figure 1A. In the control (CNT) group, the germination percentage increased rapidly, reaching a plateau (≈95%) in 24 h when compared with the other treatments, for which seeds germinated more gradually. Figure 1B shows that the GRP values of the control and the treatment with 137 mM NaCl were 95 and 85.5%, respectively. These two values showed a significant difference with respect to the GRPs obtained treating seeds with 274 mM NaCl (56.5%) and especially with the 343 mM NaCl-treated group (27%). GRPs obtained at the two highest salt concentrations were 9% and 1.5%, respectively. 

As shown in Figure 1C, MGT values of the control and the treatment with 137 mM NaCl showed significant differences in comparison with the other treatments. A similar trend was observed for GSPs, as shown in Figure 1D.

### 2.2. Seedling Development

**Figure 2:**
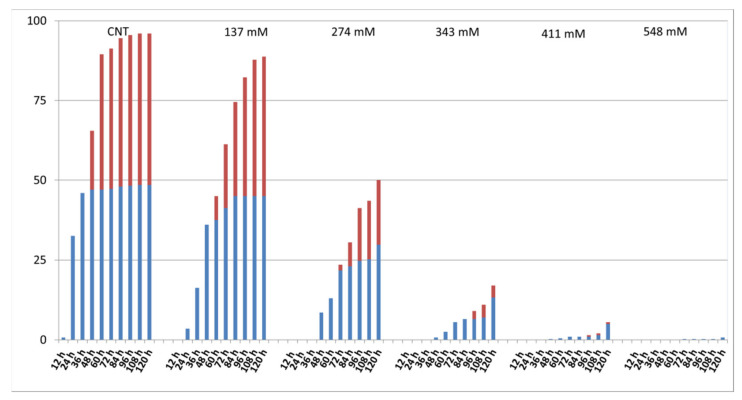
Effect of salt on the percentage of developed roots (blue) and cotyledons (red) at each time step.

Figure 2 shows the percentage of root and cotyledon appearance in the different salt treatments. Root development was delayed in all salt treatments when compared to the control group where roots appeared after 12 h. In the NaCl 137 mM group, 3.5% of roots developed after 24 h, while in the groups treated with 274 mM, 343 mM, and 411 mM of NaCl, after 48 h, 8.5%, 0.75%, and 0.25% of roots appeared, respectively. At the higher concentration, only 0.25% of roots appeared after 72 h. Regarding cotyledons, in the 137 mM NaCl samples, cotyledons appeared after 48 h, as in the control, although in different percentages. In the groups treated with 274 mM, cotyledons emerged after 72 h, while in the groups treated with 343 mM and 411 mM of NaCl, the cotyledons emerged only after 96 h. No cotyledons were visible at the higher concentrations.

### 2.3. Root and Stem Length

**Figure 3:**
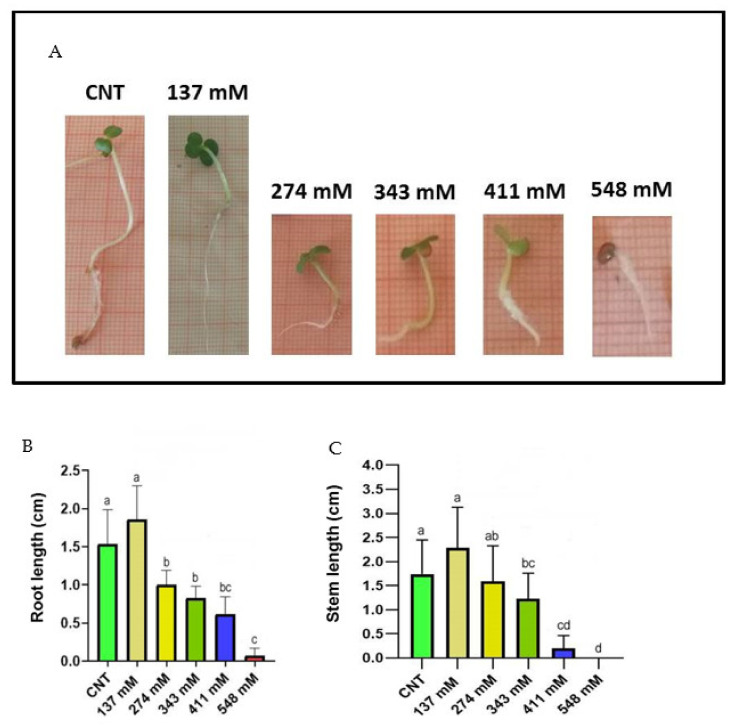
(**A**) Photographs of the seedlings grown at different salt concentrations in comparison with the control. Effect of NaCl on the lengths of both root (**B**) and stem (**C**) in *E. sativa* seedlings collected after 120 h. Different letters indicate significant statistical difference between groups (*p* < 0.05).

Root length decreased significantly with the increase in salt concentration, except for the 137 mM NaCl treatment group, where the value was not statistically different with respect to that of the control group (Figure 3A,B). 

Regarding stem length values, both control and 137 mM NaCl-treated groups showed significant differences compared to the other tested concentrations (Figure 3A,C). The highest values of the stem length were recorded in the control and 137 mM NaCl groups, whilst for the 548 mM NaCl group, it was impossible to carry out the measurement due to the lack of developed stems at this treatment.

Table 1 shows no statistical differences for the salt inhibitory effect between root and stem, except the NaCl concentration of 274 mM, where the root length was significantly more inhibited with respect to the stem length. Moreover, no inhibition was observed for the salinity level of 137 mM.

### 2.4. Fresh and Dry Weight

**Figure 4:**
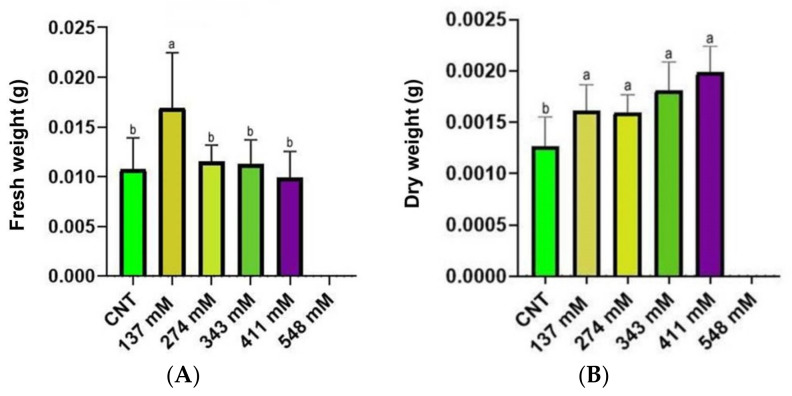
The FW (**A**) and the DW (**B**) of seedlings under salt stress compared with the control. Different letters indicate significant statistical differences between groups (*p* < 0.05).

The fresh weight (FW) of seedlings is reported in Figure 4A. No significant difference was found among control and treated samples, except for the 137 mM group that exhibited the highest value. Conversely, the seedling dry weight (DW) (Figure 4B) showed an increase in the values with the rise of salt stress level.

### 2.5. Effect of NaCl on the Lipid Aggregates of the Mesophyll

**Figure 5:**
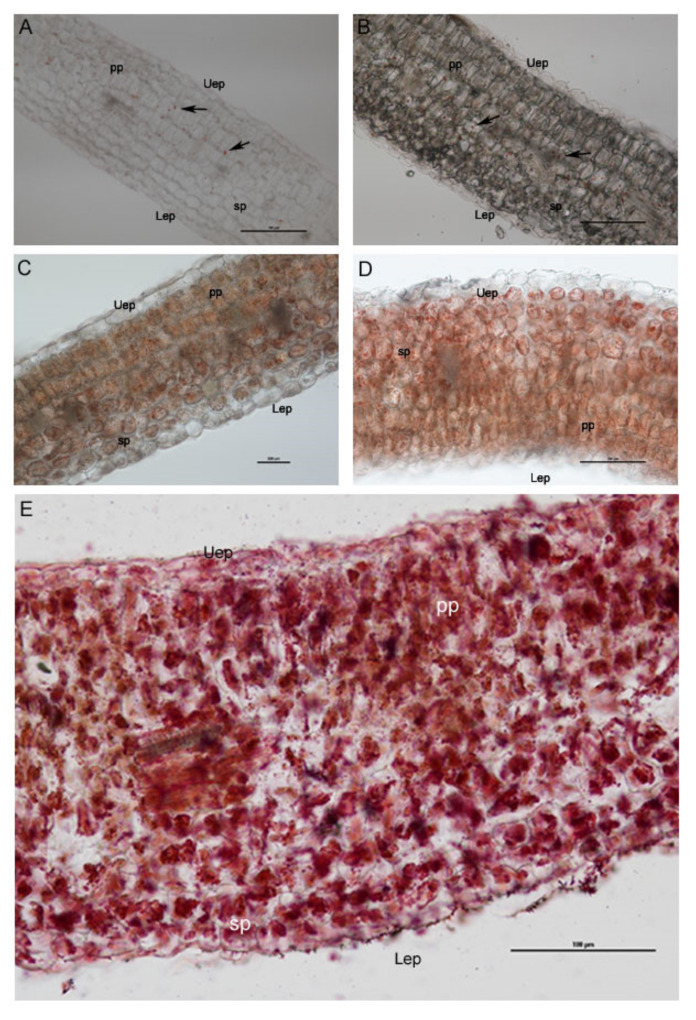
Light microscopy images showing the cross-sections of *E. sativa* mesophyll, revealing an increasing amount of the lipid content (stained in red with Sudan III–IV) in seedlings treated with 137 mM NaCl (**B**), 274 mM (**C**), 343 mM (**D**), and 411 mM (**E**) compared with the control group (**A**). Uep: upper epidermis; Lep: lower epidermis; Pp: palisade parenchyma; Sp: spongy parenchyma. Black arrows indicate isolated lipid droplets.

In the mesophyll cross-sections stained with Sudan III–IV (Figure 5), an increase in the surface of the cell section occupied by lipid bodies was observed in samples treated with the highest saline conditions (274 and 411 mM NaCl). The sections of the control group did not show lipid accumulations, except for a few droplets within the cells of the inner part of the mesophyll (Figure 5A). More numerous lipid droplets were observed in the cross-sections of the group treated with 137 mM NaCl (Figure 5B). 

Plants exposed to 137 mM and 274 mM NaCl (Figure 5B,C) treatments showed an evident increase in lipid droplets with respect to the control plants and groups. In sections from plants treated with 343 mM NaCl and 411 mM NaCl (Figure 5D,E), a great amount of lipid droplets in mesophyll was recorded. Moreover, the spongy tissue showed cells with particularly large sized lipid droplets (Figure 5C,D) that became red/violet through Sudan III–IV staining.

**Figure 6:**
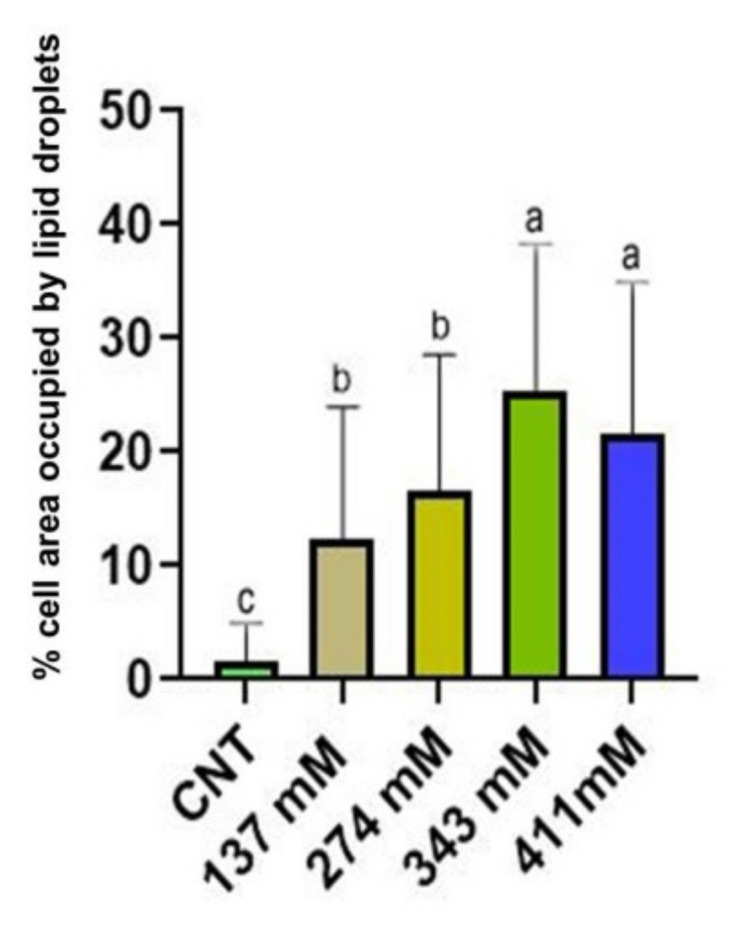
Lipid droplets (percentage of the area occupied within the cell) of the mesophylls of plants treated at 137 mM, 274 mM, 343 mM, and 411 mM NaCl compared with the control group. Different letters indicate significant statistical differences between groups (*p* < 0.05).

Statistical analysis (Figure 6) revealed a significant increase in lipid droplets for the seedlings grown under salinity conditions. Moreover, statistical differences were noted among the different treatments. Indeed, the two groups of plants grown at the highest salt levels showed the highest percentage of cell section occupied by lipid droplets. 

### 2.6. Mesophyll Intercellular Spaces and Thickness 

**Figure 7:**
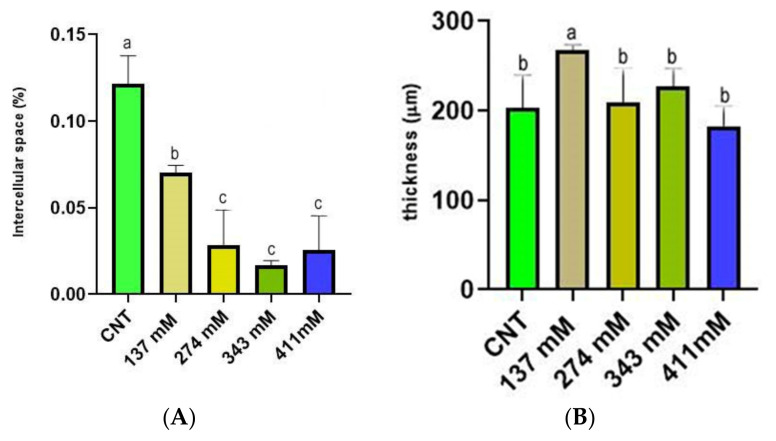
(**A**) Intercellular space (percentage of the area occupied by the section) of the mesophylls of plants treated at 137 mM, 274 mM, 343 mM, and 411 mM NaCl compared with the control group. (**B**) Mesophyll thickness of cotyledons of plants treated at 137 mM, 274 mM, 343 mM, and 411 mM NaCl compared with the control group. Different letters indicate significant statistical differences between groups (*p* < 0.05).

The percentage of intercellular spaces in the control plants was 0.12%, while already in plants treated with 137 mM NaCl solution, a drastic reduction of this parameter was observed. In particular, in the 137 mM NaCl group, the occupied area was 0.07%, and in the three higher concentrations, the intercellular area was under 0.03% (Figure 7A). Interestingly, in the 137 mM NaCl group, the mesophyll thickness of plants’ cotyledons was significantly higher in comparison to the CNT group and the other treatments (Figure 7B). 

**Figure 8:**
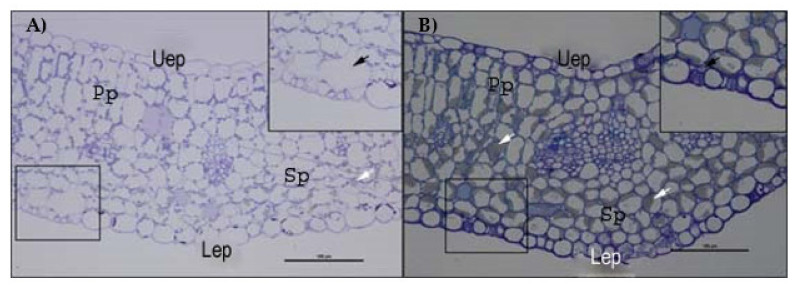
Semithin sections of *E. sativa* mesophyll stained with toluidine blue observed by optical microscopy. (**A**) Mesophyll of control plants with magnification of a substomatal chamber. (**B**) Mesophyll of plants treated with 274 mM NaCl solution with magnification of a substomatal chamber. Uep: upper epidermis; Lep: lower epidermis; Pp: palisade parenchyma; Sp: spongy parenchyma. White arrows indicate lipid aggregates; black arrows indicate the substomatal chamber.

Moreover, observing the semi-thin sections of cotyledon mesophyll, a significant reduction of intercellular spaces was noted in salt-treated samples. In particular, a drastic reduction of the area occupied by the substomatal chamber of the spongy tissue was observed (Figure 8A,B). 

### 2.7. TEM Observations

**Figure 9:**
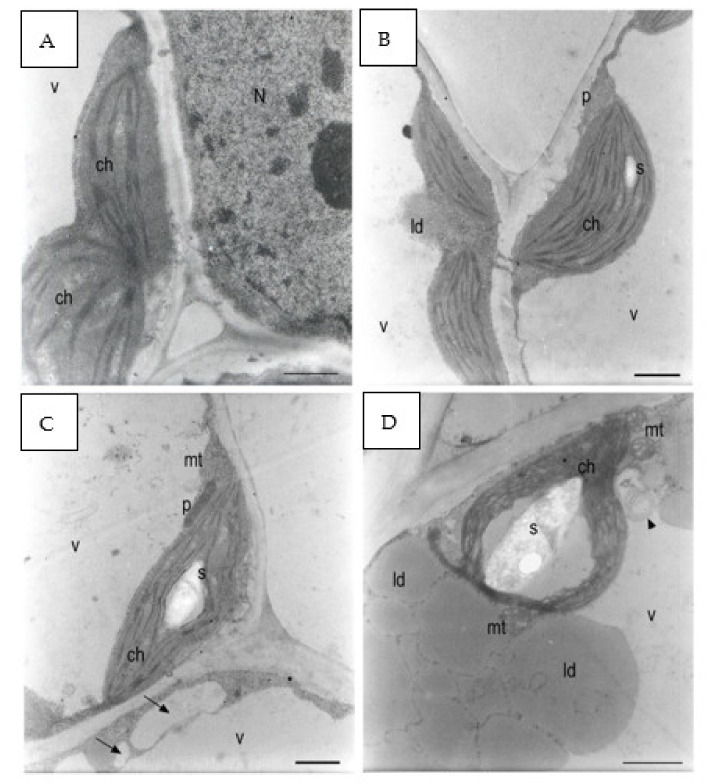

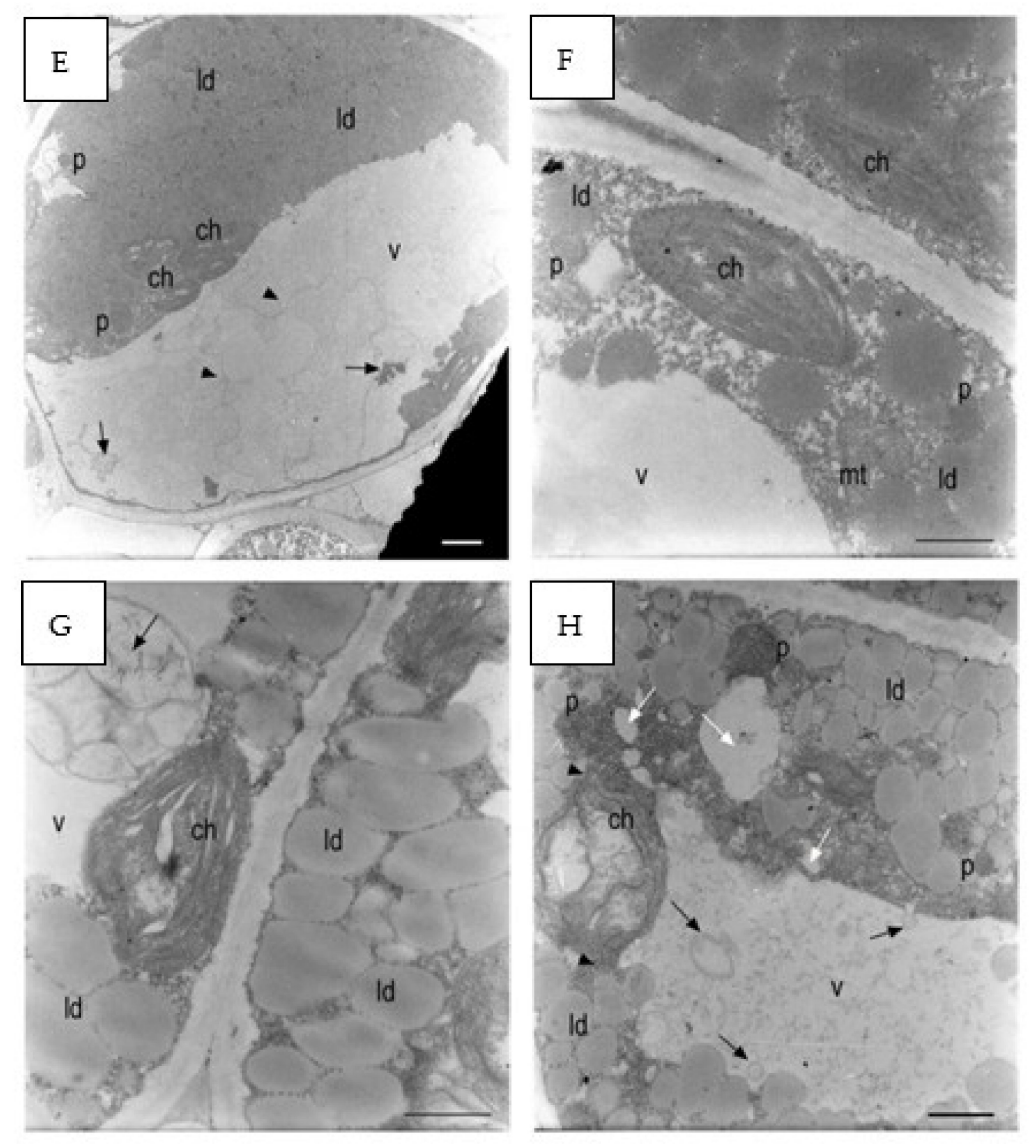
*E. sativa* seed cotyledon parenchyma (**A**). Control sample after 120 h from seeding. The plastids had very little starch. The grana were formed. (**B**) Control sample after 120 h from seeding. A small starch grain can be observed in one of the plastids. A peroxisome was positioned between two chloroplasts. Plasmodesmata connected parenchyma cells. (**C**) The 137 mM NaCl sample after 120 h from seeding. A large starch grain can be observed inside the plastid. Small- and medium-sized vacuoles (black arrows) were forming in the cytoplasm belt between the main vacuole tonoplast and the plasma membrane. (**D**) The 137 mM NaCl sample after 120 h from seeding. A large starch grain was in the plastid. Two mitochondria showed dilated cristae. Large lipid droplets can be observed between the plasma membrane and the tonoplast. Multilamellar bodies were forming in the main vacuole (black arrowhead). (**E**) The 274 mM NaCl sample after 120 h from seeding. A parenchyma cell was occupied by lipid droplets, chloroplasts not containing starch grains, and a large vacuole containing many membranes residuals (black arrowheads). (**F**) The 274 mM NaCl sample after 120 h from seeding. The plastids did not contain starch grains. Lipid droplets can be observed in the cytoplasm. The cytoplasm contained many granules. (**G**) The 411 mM NaCl sample after 120 h from seeding. The plastids contained dilated thylakoids and no starch granules. A degraded organelle with large internal vesicles was in the main vacuole (black arrow). (**H**) The 411 mM NaCl sample after 120 h from seeding. A partially degraded chloroplast had large spaces between the thylakoids that did not form grana anymore. Many lipid droplets surrounded many dark organelles with an electron-dense granular content. Microvacuoles (white arrows) were present. The vacuole was full of small particles and larger vesicles (black arrows). N: nucleus; ch: chloroplast; mt: mitochondria; p: peroxisome; v: vacuole; ld: lipid droplet s: starch. Scale bars: (**A**–**D**,**F**–**H**) 1 µm, (**E**) 2 µm.

In the control group, chloroplasts were adjacent to the plasma membrane, and the morphology of their section was oblong with well-developed grana and thylakoids (Figure 9A,B). The starch grains were in very low quantity (Figure 9B). 

Plants grown with 137 mM NaCl showed chloroplasts with starch grains in higher quantity and dimensions with respect to the control (Figure 9C). The thylakoids appeared slightly swollen around large starch grains (Figure 9D). At 274 mM, some chloroplasts were no longer adjacent to the plasma membrane and were in some points surrounded by lipid droplets and were in contact with the tonoplast (Figure 9E). The chloroplasts had highly swollen thylakoids and no more well-visible grana. The stroma was highly electron dense, and spherical aggregates were detected (Figure 9F).

At 411 mM NaCl, starch grains were absent, while lipid droplets in the cytoplasm were abundant (Figure 9G). A residual of an organelle with swollen internal vesicles was recognized in the vacuole (Figure 9G). Many electron-dense peroxisomes were able to be identified among the lipid droplets. Small vacuoles started to form in the cytoplasm, and the main vacuole contained a large amount of granular material and apparently vesicles and membranes (Figure 9H). In some points of the cells, chloroplast protrusion/vesiculation was observed.

**Figure 10:**
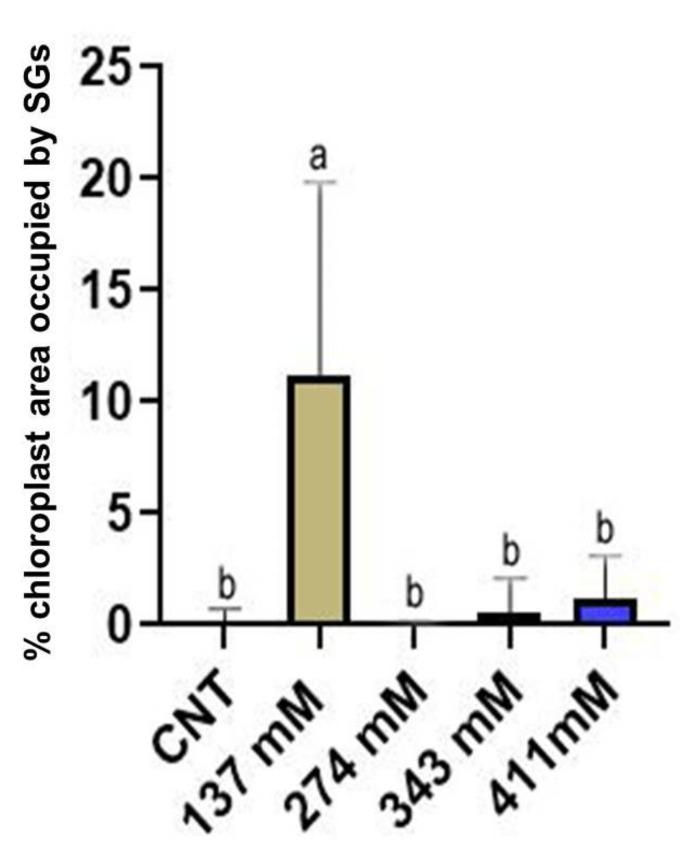
Percentage of the area occupied by starch grains (SGs) within the chloroplast of the mesophylls of plants treated at 137 mM, 274 mM, 343 mM, and 411 mM NaCl compared with the control group. Different letters indicate significant statistical differences between groups (*p* < 0.05).

Statistical analysis on the percentage of the area occupied by starch grains within the chloroplast in mesophyll cells of cotyledons was performed (Figure 10). A significantly higher occurrence of starch grains was observed in the chloroplast of the seedlings grown under the 137 mM salinity level with respect to the control group. No differences were noted between the other treated groups and the control group.

## 3. Discussion

The results revealed that germination was strongly delayed by the different salt concentrations, excluding the lowest one of 137 mM (Figure 1). Similar findings were obtained in two previous studies on *E. sativa* germination under salinity conditions [13,31]. Moreover, seed germination percentage decreased with increasing NaCl concentration as well (Figure 1B). Comparable results were reported by Aliu et al. [33] on maize varieties, showing a significant germination reduction starting from 200 mM NaCl. A reduced germination percentage starting at a salt level over 100 mM was also recorded by Zhang et al. [34] for halophytes from the Amarantaceae family, such as Bassia dasyphylla and Chenopodium rubrum. Following Khan classification [35], our results identified *E. sativa* as “marginally tolerant” since the seeds can germinate at a salt concentration between 125 and 500 mM. In addition, for the first time to our knowledge, we found the concentration of 548 mM as the salinity level beyond which the seed germination of *E. sativa* is totally inhibited. Actually, this salt concentration simulates the salinization of some coastal soils in India during the dry season, confirming that this level of salinity totally inhibited seed germination, affecting agriculture [36].

The root development was delayed with the increase in salt concentration (Figure 2). The recorded delay and inhibition of cotyledon and root development can be explained as a consequence of a reduced water uptake due to the high osmotic potential and/or by Na^+^ and Cl^−^ toxicity, in agreement with Abogadallah and Quick [37], Amiri et al. [12], and Fallahi et al. [13].

Regarding plant development, the root elongation was negatively affected by salt stress above 274 mM, whereas shoot length was significantly reduced only over 343 mM (Figure 3). Comparing the salinity effect on shoot and root at the same salt concentration, the two organs appeared to be inhibited at the same intensity (Table 1). Roots are reported to suffer the direct contact with salt, thus affecting in a negative way the enzyme activity and the cell division in root tips [38,39], while the shoot is known to be affected by the translocated Na^+^ and Cl^−^ ions from the root and the reduced water translocation [40,41,42]. 

Salt stress induced a reduction in the fresh weight, except for the concentration 137 mM where an increase in the seedling weight was observed (Figure 4A). This increase in fresh weight could have been generated by the accumulation of salt ions, leading to an increase in water uptake, as observed by Gzik [43] and Millford et al. [44]. Probably, the increase in fresh weight was not shown at a higher salinity level due to excessive salt accumulation, levels toxic enough to impair water uptake [35,45]. Regarding the dry weight, a significant increase was observed in all the NaCl-treated groups in comparison to the control (Figure 4B). This result was shown also by other authors [38,46,47] in plants such as Vicia faba, Atriplex halimus subsp. Schweinfurthii, and Cucumis sativus subjected to salinity, suggesting high ion uptake as the cause of dry weight increment. Conversely, other studies reported a decrease in fresh and/or dry weight in plants exposed to different salinity levels [48,49,50,51]. A possible explanation of the increased dry weight, worth studying in future research, could be the increment of lignification observed by some authors [52,53,54] in plants under salinity conditions.

In light of these findings, a possible stimulatory effect of salt in the low-dose zone can be hypothesized at least in the first stages of seedling development. A paradoxical effect of increase in biomass with low NaCl concentrations was observed also in other plants, such as Eucaliptus camaldulensis, Dalbergia sissoo, Sesbania grandiflora, and Casuarina spp. [55,56,57]. Those authors hypothesized that NaCl stress stimulated the rate of photosynthesis at low salt concentrations, probably due to an increase in the internal surface area per unit leaf area in succulence conditions, resulting in a higher CO_2_ absorption, as reported by Shannon et al. [58]. 

The structural integrity of the mesophyll was apparently not altered by salinity treatments (Figure 5 and Figure 8). The basic anatomy was preserved, with cotyledons showing apparently healthy palisade cell layers and spongy parenchyma. Regarding light microscopy observations, a salt-induced decrease in the intercellular space was reported (Figure 7A), especially in the spongy tissue, due to reductions in the substomatal chamber. The same phenomenon was reported in Arbutus unedo by Navarro et al. [23], who hypothesized the decrease in stomatal and mesophyll CO_2_ conductance as an effect of the intercellular space reduction generated by salt stress. In addition, the mesophyll thickness was comparable among the different salt treatments, except for the 137 mM NaCl samples, where this parameter was higher (Figure 7B). The increase in mesophyll thickness at a lower NaCl concentration (25.66 mM NaCl) was also observed by Arafa et al. [59] in sorghum plants. Strogonov [60], Nieman [61], and Rashid et al. [62] hypothesized that the higher mesophyll thickness at lower salt concentration could be due to the leaf succulence with larger cells, especially in the spongy tissue. Furthermore, the same authors supposed that at higher NaCl levels, the mesophyll thickness increase could be suppressed by salt-induced decrease in cell division. 

TEM analysis showed how salinity influenced the ultrastructure of the cotyledon mesophyll cells (Figure 9). The most affected organelle was the chloroplast, whose morphology in the treated samples showed evident alterations in comparison to the control. Moreover, three different steps in the effect of increasing salinity can be distinguished. With respect to the control, at low salinity, the chloroplasts started to store more starch grains and in a larger dimension (Figure 9C,D and Figure 10). Such behavior can be considered a consequence of a reduced sugar export capability from the cytosol with the consequent increase in plastidial starch grains, as has been observed in potato under salinity stress [63]. Another explanation could be a salt-induced inhibition of the sucrose–phosphate synthase in the cytosol. According to Salama [64], this situation can lead to a cytosolic increase in triose-phosphates, impeding a further exit of trioses from the chloroplast and hence prompting starch synthesis. On the other hand, above the NaCl concentration of 137 mM, the amount of starch in chloroplasts decreased drastically, probably because of severe impairments in photosynthesis activity [65].

Few lipid bodies within the cytoplasm were observed under the control condition with respect to all the NaCl treatments (Figure 8A,B). An increasing trend in the amount of lipid bodies in the cytoplasm was observed with the rise of the salinity level (Figure 5). According to Alencar et al. [17] and Baranova et al. [20], our findings can be explained by a salt-induced reduction in lipid reserve mobilization in rocket seedlings. Furthermore, the lack of lipid mobilization in salt-treated plants might be generated by impairment in the peroxisome oil body degradation pathway, especially at the level of the lipase SDP1 activation mechanism. Thazar-Poulot et al. [66] showed that no mobilization of oil bodies in Arabidopsis thaliana seedlings defective in lipase SDP1 genes occurs.

The number of peroxisomes increased, and the organelles appeared highly electron dense with the rising of salinity level (Figure 9E–H), as observed also in Arabidopsis thaliana by Mitsuya [67] and attributed to the upregulation of PEX11 (PEROXIN11). When such a gene is under-expressed, the peroxisomes are few and with large dimensions, while they are in a great number and a small size in over-expression conditions [68]. 

The salt-increased number of peroxisomes, coupled to the great quantity of lipid droplets in the same samples (Figure 9E–H), could be considered an attempt to preserve the efficiency of the lipid oxidation pathway under salt stress. 

Moreover, Orth et al. [69] reported that, when Arabidopsis seedlings were grown on a sucrose-free medium, peroxisome length and abundance increased, thus supposing a higher activity of such organelles when cells are deprived of sugars. Therefore, a similar condition of a salt-reduced number of cytosolic sugars could explain the salt-increased number of peroxisomes. Peroxisomes were produced by enlargement of rough endoplasmic reticulum (RER) vesicles [70], and hence the RER was also involved in the responseto salinity stress and was therefore observed in a larger amount in the cytoplasm and even in the vacuole. In addition, TEM images revealed oil bodies to be larger and irregularly shaped (Figure 9E–H), a syndrome that has been described for mutants deficient in two oleosin genes regulating the shape and size of lipid droplets, ole1 and ole2 [71,72]. Probably, the salt induced a loss in functionality of oleosins, related also to a reduced seed germination capability, as proposed by Shimada et al. [73].

In salt-treated samples, the vacuole contained ER elements and entire organelles, identified as degenerating plastids on the basis of their dimension, since in the cytoplasm, mitochondria and peroxisomes were much smaller than the plastids, and the organelles observed in the vacuole were the size of plastids or larger (Figure 9E–H). The internalization of structures into the vacuole can suggest the need of the cells to recycle organelles that are undergoing oxidative damage [74,75]. Therefore, a higher turnover of organelles in salt-stressed plants could be due to the oxidation processes of membranes, specifically thylakoids, since lipid peroxidation is one of the main mechanisms of NaCl toxicity in plants [76,77]. The role of autophagy in recycling of organelles has been observed also for other forms of abiotic stress, such as an excessive concentrations of trace metals [78].

## 4. Materials and Methods

### 4.1. Seed Germination Set-Up

To evaluate germination responses under different salt concentrations, *E. sativa* Mill. seeds (Blumen^®^, Doha, Qatar) were treated with several saline solutions, with reference to Bakhshandeh et al. (2019) [31], at the following concentrations: 0.8% *w*/*v* (137 mM); 1.6% *w*/*v* (274 mM); 2% *w*/*v* (343 mM); 2.4% *w*/*v* (411 mM); 3.2% *w*/*v* (548 mM), in addition to distilled water (as control). 

Salt treatments were carried out for 120 h in a thermostatic chamber with a temperature of 21 °C, a photoperiod of 18/6 h (light/darkness), and light radiation of 200 µmol m^−2^ s^−1^. Before treatments, seeds of *E. sativa* were sterilized in ethanol 70% (*v*/*v*) for 20 min and washed 3 times with distilled water. Then, 25 seeds were placed in 9 cm diameter Petri dishes on a filter paper moistened with 2 mL of saline solutions and put inside the thermostatic chamber. A total of 1 ml of water was added after 48 and 96 h. Each treatment was conducted in quadruplicate.

### 4.2. Germination and Seedlings Assessment

The germination, considered as the number of seeds with a 2 mm long geotropic rootlet, was recorded every 12 h up to 120 h (5 days). At each time step, the number of germinated seeds with roots was recorded and, at a more advanced development stage, the number of seedlings with cotyledons was evaluated as well. By these data, the percentage of plants with developed cotyledons, the percentage of seeds with only emerged root, and the percentage of no germinated seeds was measured at every time step. At the end of the experiment, the developed seedlings of each treatment were collected to assess the length of stems and roots using a grid paper. Stem length was considered as the distance between the base of the cotyledons and the beginning of the root. Moreover, the inhibition of salt on root and stem length was measured using the following formula

(1 − (treated sample length/mean of control length) * 100).

The plants were weighted with a precision scale (Mettler AE 260 DeltaRange, Columbus, OH, USA) to record the fresh weight and then dried into an oven at 60 °C for 72 h to measure the dry weight.

### 4.3. Light Microscopy and Transmission Electron Microscopy

Cross-sections of fresh cotyledons of developed plants for each treatment were obtained by using a vibratome (Vibratome 1000 Plus, IMEB Inc., San Marcos, CA, USA). The cross sections had a thickness of 50 µm. 

Sections were stained with Sudan III–IV a selective hystochemical dye for neutral lipids (Lison et al. 1960) and then observed through a Leitz DM-RB “Fluo” light/fluorescence microscope (Wetzler, Germany) equipped with a digital camera (Nikon DS-L1, Tokyo, Japan) to detect the amount of lipids in the plant tissues.

Cotyledon samples for each treatment were collected and immediately fixed in 1.25% (*v*/*v*) glutaraldehyde in 0.1 M phosphate buffer (pH 6.8) and stored at 4 °C for 24 h and then fixed in 1% OsO_4_ in 0.1 M phosphate buffer (pH 6.8). Afterwards, an ethanol series dehydration was performed, followed by a propylene oxide step. Lastly, samples were embedded in Spurr’s epoxy resin [79].

A Reichert–Jung ULTRACUT ultra-microtome with a diamond knife was used to cut cross-sections about 70 nm thick that were subsequently stained with uranyl acetate [80] and lead citrate [81].

The observations were performed with a Philips 201 Transmission Electron Microscope (TEM) Koninklijke Philips N.V., Amsterdam, Netherlands at 80 kV.

Semithin cross-sections (thickness 1–5 µm) were also stained with toluidine blue 0.1% and observed with a light microscope. By image analysis, the measurement of the intercellular area, considered as the percentage of area occupied by intercellular space within the area of the whole section, was carried out, together with the measurement of the mesophyll thickness. The evaluation of lipid droplets was also carried out, calculating the percentage of area occupied by oil bodies within the cell. Lastly, the estimation of the quantity of starch in the chloroplasts was measured as the percentage of the area occupied by the starch grains within the organelle.

All the images were analyzed with ImageJ [82]. 

### 4.4. Statistical Analysis 

Germination analysis for *E. sativa* seeds was performed with GerminaQuant for R (https://flavjack.shinyapps.io/germinaquant/ accessed on 29 December 2022). Time evolution of germination under different saline treatments was assessed, and the following three germination indexes were estimated.

Germination rate percentage (GRP): defined as the percentage of seeds that germinate developing the cotyledons.

$$GRP=\left(\frac{\sum_{i=1}^{k}n_{1}}{N}\right)\times 100$$

Mean germination time (MGT) expressed in hours:

$$MGT=\left(\frac{\sum_{i=1}^{k}n_{1}t_{1}}{N\sum_{i=1}^{k}n_{1}}\right)$$

Germination speed percentage (GSP):

$$GSP=\frac{\sum_{i=1}^{k}G_{i}}{\sum_{i=1}^{k}G_{i}X_{i}}\times 100$$

where $n_{1}$ is the number of seeds germinated in the $i^{th}$ time, and k is the last day of the evaluation process for germination.

The statistical analysis of the intercellular space, stem length, and root length were performed with Graph Pad Prism (version 8.0.1) using the one-way ANOVA test (one-way analysis of variance) and the Tukey–Kramer test for multiple comparisons. 

## 5. Conclusions

**Figure 11:**
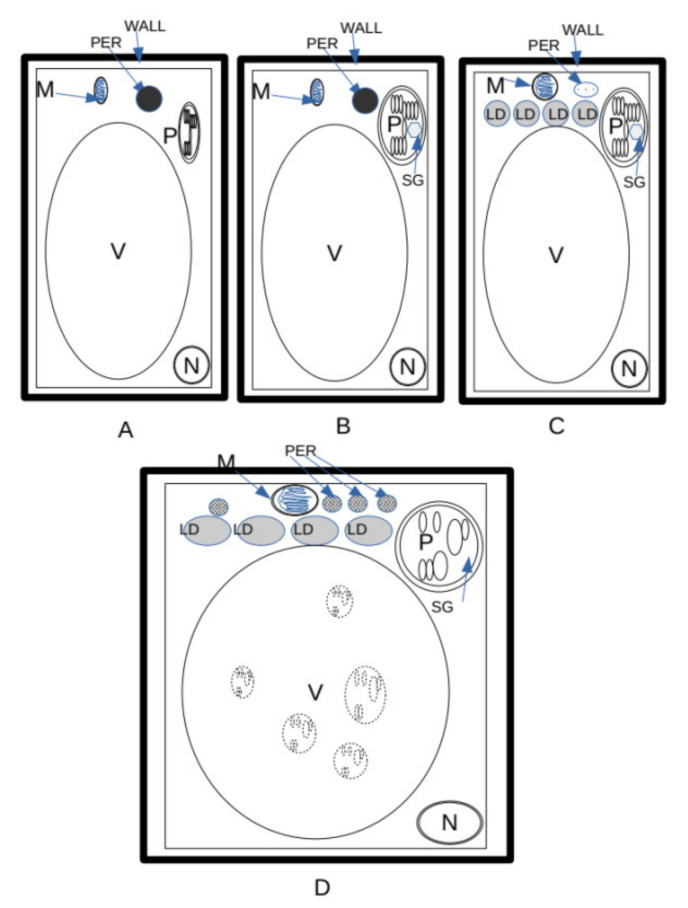
Cellular changes in *E. sativa* seedlings under growing saline concentrations. (**A**) Control. Lipid droplets (LD) were dismantled, and sugars produced with gluconeogenesis were regularly exported. Little or no starch inside the plastids was formed. (**B**) Low salinity. Accumulation of starch in chloroplasts was possibly due to a reduction in the capability of cells to export sugars (see Figure 5). (**C**) Medium salinity. Large amount of starch in chloroplasts due to a reduction in the capability of cells to export sugars. Higher number of lipid droplets with respect to (**B**), due to reduction in efficiency of the use of lipid storage. Increase in number of glyoxisomes. (**D**) High salinity. Increase in lipid droplets due to reduction in efficiency of the use of lipid storage. Increase in number of glyoxisomes. Plastids entered into the vacuole and underwent dismantling as a result of autophagy. LD = lipid droplets; M = mitochondrion; N = nucleus; PER = peroxysomes/glyoxysomes; SG = starch grain; V = vacuole; WALL = wall.

**Figure 12:**
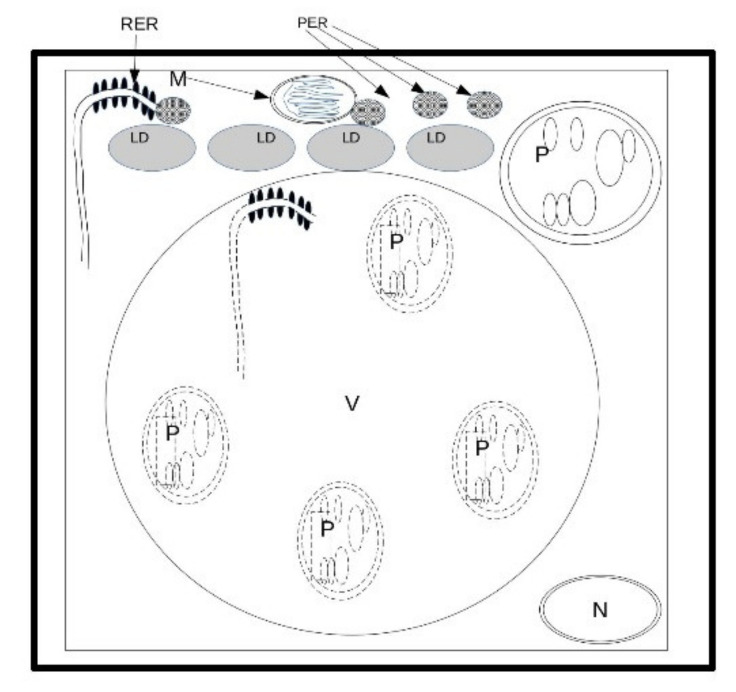
Response mechanism to salinity. Since salinity reduces the capability to use lipids in lipid droplets, cell adaptation is apparently based on an increase in peroxisome number and hence in RER activity in the production of peroxisomes. The resistance to salt stress is apparently based on an increase in autophagy recycling of plastids and RER in the vacuole. LD = lipid droplet; M = mitochondria; N = nucleus; P = plastid; PER = peroxisomes; RER = rough endoplasmic reticulum; V = vacuole.

*E. sativa* showed the capability to germinate until a salinity level of 548 mM. Moreover, in comparison with the control (Figure 11A), the lower level of salinity (Figure 11B) stress caused symptoms ranging from diminished growth at the macro-morphological level to changes in starch accumulation in plastids and (at a higher concentration) an increase in lipid droplets in the developing cotyledons (Figure 11C). It is probable that the capability of peroxisomes to interact with lipid bodies decreased under salinity conditions, and hence the cell produced more peroxisomes to counterbalance this effect. Intriguingly, a low concentration of salt, not overtaking 137 mM, seemed to have a stimulatory effect on seedling development. Lastly, salt stress led to the activation of autophagic processes in *E. sativa* seedlings, thus ensuring the recycling of damaged organelles and cytosolic components (Figure 11D). The response mechanism to salinity is based on the capability to use lipids in lipid droplets, and, consequently, on an increase in peroxisome number and RER activity in the production of peroxisomes themselves (Figure 12). The adaptation to salt stress is apparently based on an increase in autophagic recycling of plastids and RER in the vacuole (Figure 12).

## Figures and Tables

**Table 1:** Comparison of the inhibitory effect of NaCl treatments as a percentage of both root and stem in *E. sativa* seedlings collected after 120 h. Different letters indicate significant statistical differences between groups (*p* < 0.05).

Salt Treatment	% Root Inhibition	% Stem Inhibition	R	*p*-Value
NaCl 0.8% (137 mM)	−21.09 ^a^ ± 10.1	−30.95 ^a^ ± 17.31	0.0170	0.1782
NaCl 1.6% (274 mM)	34.95 ^b^ ± 4.4	8.80 ^a^ ± 14.99	0.1666	0.0045
NaCl 2% (343 mM)	52.30 ^b^ ± 6.77	29.40 ^a^ ± 10.79	0.1845	0.2773
NaCl 2.4% (411 mM)	59.87 ^b^ ± 7.47	77.21 ^a^ ± 9.38	0.3214	0.2407
NaCl 3.2% (548 mM)	90.25 ^b^ ± 3.251	100 ^a^ ± 0	0.8182	0.0955

## Data Availability

Not applicable.
